# “Envbiotics”—a concept based on host immune-gut environment and microbiota balance: expanding and refining the concepts of prebiotics and postbiotics

**DOI:** 10.3389/fmed.2025.1750262

**Published:** 2026-01-15

**Authors:** Ming-chen Cao, Fan-bo Jing, Wen-xiao Wang, Ze-nan Zhang, Yun-yan Shi, Wen-jing Li, Yan-lin Shao, Hong-xia Yu, Xiao-min Xing

**Affiliations:** The Affiliated Hospital of Qingdao University, Qingdao, China

**Keywords:** Envbiotics, gut microecosystem, gut-brain axis, intestinal microecology, microbiota

Current approaches to understanding gut microbiota largely concentrate on improving host health by fine-tuning the composition and quantity of microbial populations. Prebiotics primarily address compounds that act as direct substrates, energy sources, or growth stimulators for select gut bacteria (1). Postbiotics, on the other hand, focus on the cumulative effects of microbial activity and their metabolic derivatives (2). Despite their significance in fostering microbiota balance, these models often overlook the crucial role of non-nutritional environmental factors in shaping microbial processes, thereby lacking comprehensive theoretical frameworks.

While the latest international expert consensus on prebiotics, published in Nature Reviews Gastroenterology & Hepatology, recommends expanding the definition to include compounds like polyphenols and fatty acids that can be metabolized by gut microbiota, thus broadening the category beyond dietary fiber components, we contend that this revised approach still has certain limitations. Specifically, it continues to not fully account for how non-nutritional elements within the gut indirectly support the growth and colonization of beneficial microbes. Such mechanisms may involve enhancing microbial resilience against external perturbations (3).

The interactions between the gut microbiota and the intestinal environment are dynamic and context-dependent, varying significantly under different physiological states of the host. Consequently, microbiota supplementation or microbiota transplantation alone constitutes an insufficient approach. For example, research published in Cell indicates that stress may suppress neural activity in the central nucleus of the amygdala, consequently attenuating vagal activity (4). This attenuated vagal activity is hypothesized to reduce mucin secretion from Brunner's glands in the duodenum. The subsequent decrease in mucin may, in turn, impair the proliferation of *Lactobacillus*, potentially contributing to increased intestinal permeability and compromised immunity, which will further exacerbate dysbiosis of the gut microbiota (4). And acupuncture is known to act on the vagus nerve, to regulate digestive gland function, and to influence the abundance and metabolic patterns of the gut microbiota by affecting host immune status and the expression of endogenous factors (5). Coincidentally, contrary to its beneficial reputation, a high abundance of *Akkermansia muciniphila* in the gut exacerbates radiation-induced intestinal injury by initiating a deleterious vicious cycle. During acute radiation-induced intestinal injury, *Akkermansia muciniphila* activates mucin metabolism genes, leading to a compromised mucosal barrier that promotes bacterial adhesion and translocation. This event drives the polarization of intestinal stromal macrophages toward a pro-inflammatory phenotype, secreting cytokines like IL-6 and TNF, which subsequently suppress the intestinal stem cell- transit-amplifying (TA) cells -goblet cell axis and inhibit mucin secretion, thus amplifying the initial damage (6). Furthermore, autologous microbiota transplantation, for instance, appears more effective at reinstating functional microbial communities compared to allogeneic transplantation, which often struggles to maintain conditions mirroring the donor's microbial environment following external interventions (7). Moreover, the microbiota's metabolic outputs are inherently shaped by its intestinal surroundings (8). For example, XBP1 ablation leads to diminished mucin 2 (MUC2) expression and impaired mucus layer formation in the colon; the combined loss of XBP1 and caspase-8 can result in bacterial translocation from the lumen into the subepithelial layer, triggering systemic inflammation (9). Similarly, another study reveals a novel mechanism by which commensal bacteria utilize host-derived FABP2 to drive dysbiosis and worsen Crohn's disease pathology (10). These host-derived proteins, such as MUC2 in the mucus layer and FABP2, are prime examples of the non-nutritional, structural, and regulatory components of the intestinal microenvironment that profoundly shape microbial behavior and host-microbe interactions. In summary, improving the gut microenvironment for microbial proliferation by enhancing the host's physiological state, ultimately achieving the restoration or optimization of the host's gut microbiota structure and function, may become a significant future research direction.

As shown in Table 1, unlike the concepts of prebiotics and postbiotics, the concept of “Envbiotics” includes various external and internal biological influences, energies, or conditions that are not directly utilized by gut microbiota for acquiring carbon, energy, or nutrients. It is known that many non-antibiotic drugs and those not excreted through the gut are typically not utilized or metabolized by the gut microbiota. However, they can still reshape the gut ecosystem by affecting the host's physiological state, thereby influencing the gut microbiota. A recent study published in mSystems by the American Society for Microbiology analyzed fecal samples from over 2,500 participants. It found that common medications such as antidepressants, antipsychotics, beta-blockers, proton pump inhibitors, and benzodiazepines can cause significant alterations in the gut microbiota. These changes persist long after discontinuation, and the gut microbiome exhibits predictable fluctuations when specific medications are added or removed (11). Similarly, a recent study published in Nature found that immunosuppressants, heart medications, blood pressure drugs, and antidepressants may also act as “accomplices” in increasing the risk of intestinal infections (12).

**Table 1:** The primary mechanism and target of Prebiotics, Postbiotics and Envbiotics.

**Comparison dimension**	**Prebiotics**	**Postbiotics**	**Envbiotics**
Definition	A substrate that is selectively utilized by host microorganisms conferring a health benefit.	A preparation of inanimate microorganisms and/or their components that confers a health benefit on the host.	Refers to a class of exogenous or endogenous bioactive factors, energy, or conditions that are not directly used by gut microbiota as carbon sources, energy, or nutrients.
Mechanisms of action	(1) Act as direct substrates, energy sources, or growth stimulators for select gut bacteria; (2) metabolite-mediated: beneficial bacteria metabolize prebiotics to produce short-chain fatty acids, indirectly exerting anti-inflammatory and barrier function-regulating effects.	(1) Direct immunomodulation: induces regulatory T cells to secrete IL-10 and inhibits the NF-κB inflammatory pathway; (2) antibacterial effect: bacteriocins directly inhibit pathogenic bacteria; (3) barrier enhancement: short-chain fatty acids (e.g., butyrate) promote the repair of intestinal epithelial cells.	(1) Optimize the gut microecological environment: regulates intestinal pH to inhibit pathogenic bacteria and repair the mucus layer structure; (2) indirect microbiota regulation: promotes the colonization of beneficial bacteria by optimizing ecological niches rather than directly providing nutrition; (3) host-microbiota interaction regulation: modulates host immune status and improves intestinal barrier function.
Target objects	(1) Specific beneficial microorganisms in the intestine (e.g., *Bifidobacterium, Lactobacillus*); (2) microbiota metabolic pathways.	(1) Host immune cells (macrophages, dendritic cells); (2) anti-inflammatory and antioxidant effects on intestinal epithelial cells, macrophages, and neutrophils.	(1) Intestinal microecological environment (core target); (2) overall endogenous microbiota (not specific strains, emphasizing the coordination of microbiota structure and function); (3) host intestinal barrier; (4) host immune system (mucosal immune cells, systemic immune factors).
Scope of coverages	(1) Carbohydrates: oligosaccharides containing a small number (3–10) of simple monomers to longer chain lengths (up to about 60 monomers) that might be mostly linear; (2) non-carbohydrates: compounds like polyphenols and fatty acids that can be metabolized by gut microbiota.	(1) Metabolites: Short-chain fatty acids, bacteriocins, vitamins; (2) cellular components: exopolysaccharides, cell wall fragments, bacterial lysates.	(1) Physical factors: intestinal temperature, osmotic pressure, spatial arrangement of the mucosal layer; (2) chemical factors: pH, redox potential, bile salt/short-chain fatty acid concentration; (3) biological factors: host mucus secretion volume, antimicrobial peptide levels, immune cell function.
Dependence on “endogenous microbiota”	Relies on exogenous substrate supply to regulate endogenous microbiota, requiring the metabolic capacity of specific microbiota.	Does not directly affect the structure of endogenous microbiota, but indirectly influences microbiota activity through metabolites or cellular components of intestinal microbiota.	Takes “optimizing the structure and function of endogenous microbiota” as the core goal, emphasizing the utilization of the potential of the host's own microbiota and avoiding the problems of “difficult colonization and activity differences” of exogenous probiotics.

Besides that, Polygonatum cyrtonema polysaccharides (PCP) significantly reduced the symptoms of colitis by directly modulate the inflammatory immune response and intestinal barrier function, the alleviating effects on colitis are independent of gut microbiota, which in turn regulates gut microbiota. Oral PCP administration markedly inhibited excessive inflammation-mediated immune response by modulating inflammatory cytokines secretion and Th17/Tregs cell balance, restored IgA, ZO-1, Occludin, and MUC2 expression to enhance intestinal barrier function and restored gut microbial composition (13). Futhermore, pyrroloquinoline quinone (PQQ), widely present in prokaryotes, plants, and mammals, is an aromatic quinone compound with physiological functions similar to vitamins. This compound is primarily synthesized in nature by specific bacterial groups, including Gram-negative bacteria such as *Klebsiella pneumoniae* and *methylotrophic bacteria*, etc. (14). Notably, these bacterial species are typically classified as opportunistic pathogens or commensal microbiota within the human gut, which produced by pathogenic bacteria may not be suitable for classification within the category of postbiotics, their classification is relevant for understanding PQQ's selective effects on the gut microbiome. As a redox enzyme cofactor with broad biological activity, theoretically, it does not possess selective promotion or inhibition effects on specific types of microbial communities. However, empirical studies indicate it can selectively increase the abundance of beneficial bacteria (14, 15). This selectivity may arise because PQQ primarily exerts its “Envbiotics” effects, by regulating the host's immune status and the overall gut microenvironment. We hypothesize that this immunomodulatory action and microenvironmental sculpting, rather than direct indiscriminate bacterial promotion, underpin the observed selective enrichment of beneficial taxa. Next but not last, Exosome- and vesicle-like components have been shown to regulate gut microbiota abundance and metabolism, often indirectly through their influence on host conditions such as inflammatory bowel disease (16). These biomolecular complexes, comprising RNA, mRNA, proteins, and other macromolecules, are primarily internalized by host immune cells to exert their regulatory effects, rather than being directly metabolized by the gut microbiota. Consequently, they cannot be simply categorized as prebiotic systems based on chemical structure. For example, Chinese plum-derived PM-EVLPs demonstrate anti-colitis effects and improve gut microbiota composition by suppressing NLRP3 inflammasome activation through miR-159-mediated blockage of NEK7-NLRP3 interactions (17).

At the United European Gastroenterolog (UEG) Week academic conference held on October 5, 2025, in Berlin, Germany, Professor Chloé Melchior from the University of Rouen, France, reported that a composite formulation combining xyloglucan and pea protein can persistently reside in the intestinal mucosal layer through a synergistic mechanism. This forms a stable mechanical barrier that significantly enhances intestinal barrier function and alleviates visceral hypersensitivity, thereby regulating gut microbiota homeostasis. As a non-pharmaceutical intervention, it offers a valuable new therapeutic option (18). Such observations, where non-nutritional factors profoundly alter the gut milieu, point to a broader regulatory paradigm that ultimately aims to optimize the host's native microbiota's structure, composition, and functionality to enhance overall host health. This paradigm is encapsulated by the term “Envbiotics,” which emphasizes environmental modulation as a distinct regulatory pathway from direct microbial structural modification, population control, or nutrient supply, thus bridging gaps in current theories about non-nutritional regulatory mechanisms. Envbiotics focuses on the environmental conditions critical for microbiota survival and performance, including physicochemical factors (e.g., intestinal acidity, oxidation-reduction potential, osmotic balance, temperature, ion concentrations, bile salts, short-chain fatty acids), spatial aspects (e.g., arrangement of the intestinal mucosal layer), and biological markers (e.g., volume and composition of host mucus secretion, levels of antimicrobial peptides, immune cell functions, presence of other symbiotic organisms). The concept of “Envbiotics” highlights the environment as a central factor in microbiota modulation, offering new insights into non-nutritional, indirect microbial influences that effectively regulate gut microbiota and contribute to host health.
